# Effects of Prickly Ash Seed Dietary Supplementation on Meat Quality, Antioxidative Capability, and Metabolite Characteristics of Hu Lambs

**DOI:** 10.3390/foods13213415

**Published:** 2024-10-26

**Authors:** Qiao Li, Yi Wu, Xingcai Qi, Zilong Liu, Chunhui Wang, Xueyi Ma, Youji Ma

**Affiliations:** 1 College of Animal Science and Technology, Gansu Agricultural University, Lanzhou 730070, China; liqiao1109@163.com (Q.L.); wy1593050417@126.com (Y.W.); 18794226760@163.com (X.Q.); lzl107332202076@163.com (Z.L.); chwang5522@163.com (C.W.); maxueyi0303@163.com (X.M.); 2 Gansu Key Laboratory of Animal Generational Physiology and Reproductive Regulation, Lanzhou 730070, China

**Keywords:** prickly ash seeds, Hu sheep, meat quality, antioxidant status, metabolomics

## Abstract

In China, the processing of prickly ash (PA) produces a large number of by-products, including prickly ash seeds (PASs), which are rich in bioactive components such as flavonoids and phenolic compounds, and which may have an important influence on meat quality and muscle metabolites. Therefore, this study aimed to assess the impact of dietary PAS supplementation on the meat quality, antioxidant activity, and metabolite characteristics of lambs. Eighteen 3-month-old Hu lambs (25.66 ± 3.03 kg body weight) were randomly allotted to three different dietary treatment groups. In the three dietary treatments, 0% (basal diet, CON), 3% (CON with 3% PAS, low-dose PAS, and LPS), and 6% (CON with 6% PAS, high-dose PAS, and HPS) PASs were used. Results indicated significant improvements in the HPS group, including reduced cooking loss and increased fat content. The L* and b* 45 min values were significantly lower in the PAS groups than those in the CON group (*p* < 0.05). Additionally, dietary PAS supplementation increased in MUFA, PUFA, n-3 PUFA, PUFA/MUFA ratio, NEAA, and FFA compared to the CON group. Furthermore, PAS supplementation significantly improved serum and muscle antioxidant capacity. Metabolomic analyses revealed that increased metabolites, such as tryptophan, leucine, citric acid, adenosine 5′-triphosphate, creatine phosphate, inosine, and *α-ketoglutaric acid, were primarily enriched in the biosynthesis of cofactors and nucleotide and* purine metabolism pathways. Notably, supplementation with 6% of PASs exhibited the most prominent effect on lamb meat quality in this study. Therefore, the application of PASs as a feed component in lamb production can not only improve meat quality and muscle antioxidant capacity but also save feed costs.

## 1. Introduction

With the growth of the global population and changes in consumption patterns, animal husbandry is facing unprecedented challenges. On the one hand, traditional feed resources are facing the problem of restricted supplies, which not only affects the cost of feed but also affects the production performance and meat quality of animals. On the other hand, to ensure food safety and public health, the use of antibiotics in feed has been severely restricted worldwide, which requires us to find effective alternatives to antibiotics to enhance animal health and production efficiency [1,2]. In this context, the potential of plant-based feed has gradually received attention. Plant-based feed, including various crop by-products, food industry by-products, and specialized energy crops, not only provides abundant nutrients but may also contain a variety of bioactive substances. These substances have received extensive attention due to their role in improving animal growth performance, meat quality, and flavor [3,4]. Therefore, the development of green alternative feed and unconventional feed resources while improving animal production performance and meat quality has gradually become a research hotspot for animal nutritionists.

Prickly ash seeds (PASs) are one of the green alternative feed energy sources that are promising to alleviate feed resources. PASs are the main processing by-product of the prickly ash (PA) (*Zanthoxylum bungeanum*) industry, and their weight accounts for 60–70% of the total weight of PA fruit [5]. Despite China’s significant cultivation and use of PA, the by-products of its processing are typically treated as waste or directly discarded, with limited development and utilization [6]. Our previous study found that PASs are rich in nutrients [7] and can be used as a good alternative feed resource. In addition, PASs and their processed products contain polyphenols, flavonoids, alkaloids, and other biologically active ingredients which are widely used in the food processing industry and livestock feed field [8]. Mu et al. found in their study that the total polyphenol content of pepper seeds ranged from 72.83 to 138.84 mg and the flavonoid content ranged from 29.78 to 57.56 mg after determining the total polyphenols and flavonoids of pepper seeds by using different polarity solutions [9]. There have also been recent examples of the application of PASs in animal production. For example, in pigs, the replacement of 5% and 7.5% of corn with PASs reduced the content of SFAs and increased the content of PUFAs and n-3PUFAs of the *Longissimus thoracis* (LT) [10]. This may be due to the addition of PASs changing the α-linolenic acid content in the diet, which in turn affected its transport efficiency from the liver to the muscle tissue [11]. Tian et al. found that adding 5% PASs to the diet of Jian carp did not adversely affect growth or health status, but 7.5% PASs did have a negative impact on growth [12]. However, the impact of adding PASs to lamb feed on the body and meat quality remains unclear because of variations in nutrient digestion and absorption among fish, monogastric animals, and ruminants, so this is awaiting further investigation.

Given the scarcity of reports on PASs in the lamb industry and their biological functions, we hypothesized that the application of PASs as a feed ingredient or additive could improve lamb meat quality and antioxidant activity. Therefore, this study aims to explore the impact of dietary PASs on meat quality, antioxidant activity, and muscle metabolism in lamb meat, shedding light on their nutritional and health-promoting value.

## 2. Materials and Methods

### 2.1. Ethics Statement

The present study was approved by the Ethics Committee of the Gansu Agricultural University (GSAU-Eth- ASF2022-008).

### 2.2. Preparation of Diets

PASs were collected between August and September 2021 at planting base of prickly ash in Dongxiang Autonomous County, China. The nutritional components of PASs are presented in Supplementary Table S1. We analyzed the metabolites in PASs using high-performance liquid chromatography–mass spectrometry, and among them, flavonoids and phenols accounted for 10.97% and 4.52%, respectively (Supplementary Figure S1). The basal diet was prepared according to the “Nutrient requirements of meat-type sheep and goat (NYT816-2021)” in China (Supplementary Table S2).

### 2.3. Animals, Feeding Management, and Experimental Design

Eighteen 3-month-old male Hu lambs (25.66 ± 3.03 kg) were randomly allotted to three different dietary treatment groups, with six in each group. In the three dietary treatments, 0% (basal diet, CON), 3% (CON with 3% PAS, low-dose PAS, LPS), and 6% (CON with 6% PAS, high-dose PAS, HPS) PAS proportions were used. The addition of PASs to the LPS and HPS groups replaced an equivalent amount of roughage, ensuring that all experimental diets had the same nitrogen and energy levels. The experiment period lasted 100 days, including a 10-day adaptation period and a 90-day experimental period. During the feeding period, a total mixed ration (TMR) was provided to Hu lambs at 08:00 and 18:00 h every day with the feed supply adjusted according to 5~10% of the residual feed. All lambs had free access to food and water.

### 2.4. Sample Collection

On the 90th d, after fasting for 12 h, all lambs within a slaughter group were slaughtered. Specifically, the lambs were exsanguinated after being stunned according to standard commercial procedures. Serum samples obtained from the collected blood were stored at –20 °C for subsequent analysis. LT samples were collected within 30 min after slaughter; one sample was used for meat analysis, and the other one was frozen in liquid nitrogen and stored at −80 °C for subsequent analysis.

### 2.5. Antioxidant Enzyme Activity Assay

The glutathione peroxidase (GSH-Px, no. HY-60005), total antioxidant capacity (T-AOC, no. HY-60021), superoxide dismutase (SOD, no. HY-60001), and catalase (CAT, no. HY-60015, no. HY-60015) activities, and the content of malondialdehyde (MDA, no. HY-60003) of the serum and LT muscle were measured using ELISA kits purchased from Beijing SINO-UK Institute of Biological Technology (Beijing, China).

### 2.6. Meat Quality Analysis

The pH of the LT was determined using a portable pH meter (CP-461, Elmetron, Zabrze, Poland) at 45 min (pH _45min_) and 24 h (pH _24h_) post mortem. For color measurement, the LT was exposed to air for 45 min to allow for blooming. Subsequently, the lightness (L*), redness (a*), and yellowness (b*) were measured using a CM-2600d chromameter (Konica Minolta, Osaka, Japan). A 1 cm thick sample was cut from the central part of the eye muscle, with a circular sample of 2.532 cm in diameter, and weighed as m1. This sample was then pressed for 5 min with a tablet press under 35 kg and weighed as m2. The water loss rate (%) was calculated as [(m1 − m2)/m1] × 100%. The drip loss, cooking loss, and shear force were measured according to the previous literature [13]. To put it simply, a 100 g LT was placed in a polyethylene bag for water-bath heating until the core temperature of the LT reached 70 °C. After cooling to room temperature (23 °C), we measured meat tenderness using 1.27 cm diameter cores sampled along the muscle fiber. Six technical replicates were measured for each shear force sample, using a TA.XTplus Texture Analyzer (Stable Micro Systems, Godalming, UK). In addition, a Foss FoodScan Meat Analyzer (FoodScan Lab, FOSS, Hillerød, Denmark) was used to determine the nutritional value of LT. The amino acid was analyzed in a UPLC-MS/MS system (UPLC, ExionLC™ AD; MS/MS, QTRAP^®^ 6500+ mass spectrometer), following the procedures as outlined previously [14]. Gas chromatography (Agilent 7890) was used to measure fatty acids according to the previously described method [15].

### 2.7. Metabolomics Analysis

The experimental process of the metabolomic analysis included sample extraction and analysis, metabolite identification annotation, data quality control, and statistical analysis, all of which were performed at Wuhan MetWare Biotechnology Co., Ltd. (Wuhan, China) following their standard procedures [16]. The samples were analyzed using an LC-ESI-MS/MS system (UPLC, ExionLC AD; MS, QTRAP^®^ 6500+ System). The UPLC conditions used the parameters described above [17]. Finally, data were acquired using multiple reaction monitoring with a triple quadrupole tandem mass spectrometer. The datasets were processed using Analyst software version 1.6.3 (Applied Biosystems, Concord, Canada) and MultiQuant software version 3.0.3 (Absciex, Framingham, MA, USA). Principal component analysis (PCA) was performed using the prcomp function in R to observe the distribution among samples. Subsequently, orthogonal partial least squares discriminant analysis (OPLS-DA) was performed to analyze the data. Multivariate analysis of OPLS-DA model variable importance projection (VIP) was used to screen differential metabolites. In this study, VIP > 1, FC  ≥ 1.5, or FC ≤ 0.667 were set as criteria for differentially accumulated metabolite (DAM) screening. To study the trend of relative content of DAMs in different groups, the relative content of DAMs in the three groups was subjected to unit variance scaling treatment, followed by K-Means cluster analysis. The enrichment analysis of DAMs was performed using the KEGG database, and *p* < 0.05 was considered statistically significant.

### 2.8. Statistical Analysis

Statistical analyses were conducted using one-way ANOVA of SPSS (version 22.0; SPSS Inc., Chicago, IL, USA). The statistical differences (data on meat physicochemical characteristics and antioxidation capacity parameter) were examined via Duncan’s multiple comparison tests, and the statistical significance was considered at *p* < 0.05. The correlation between the differentiated metabolites and phenotypes was performed using Pearson correlation analysis, and Pearson correlation coefficients r > 0.5, r > −0.5, and *p* < 0.05 were considered as a strong correlation.

## 3. Results

### 3.1. Meat Physicochemical Characteristics and Meat Quality of the Hu Lambs

In the HPS group, fat content was significantly higher compared with the CON and LPS groups (*p* < 0.05). The cooking loss in the HPS group was significantly lower than that in the CON group (*p* < 0.05). The L* and b* values in the LPS and HPS groups were significantly lower than those in the CON group (*p* < 0.05). There were no significant differences in moisture, ashes, protein, collagen, drip loss, water loss rate, shear force, or pH among the three groups (*p* > 0.05) (Table 1).

### 3.2. Antioxidant Capacity of the LT Muscle

The antioxidant capacity and MDA content of the Hu lamb serum and muscle are presented in Table 2. In the HPS group, the serum exhibited significantly higher T-AOC, SOD, GSH-Px, and CAT activities, along with lower MDA content (*p* < 0.05), compared to the CON group. In the LPS group, serum T-AOC and CAT enzyme activities were significantly increased (*p* < 0.05), but other indicators showed no significant differences compared to the CON group. In muscle tissues, the HPS group had significantly higher enzyme activities for T-AOC, GSH-Px, and CAT, along with lower MDA content compared with the CON group (*p* < 0.05).

### 3.3. FA Composition

In this study, saturated FA (SFA) contents were significantly lower in the LPS and HPS groups than in the CON group (*p* < 0.05). The LPS and HPS groups exhibited a significant increase in MUFA, PUFA, n-3 PUFA, and PUFA/MUFA ratios compared to the CON group (*p* < 0.05). In addition, the n-6/n-3 ratio was significantly higher in the HPS group than in the CON group (*p* < 0.05) (Table 3).

### 3.4. Amino Acids and the Composition of Their Derivatives

The amino acid composition of the LT used in this study is shown in Table 4. Compared with the CON group, the HPS group exhibited significant increases in tryptophan, aspartate, glycine, arginine, and glutamic acid contents (*p* < 0.05), and the LPS group exhibited a significant decrease in histidine content (*p* < 0.05). Furthermore, after the addition of PAS, the content of threonine, proline, flavor amino acid (FAA), and non-essential amino acid (NEAA) was significantly increased in the LPS and HPS groups (*p* < 0.05). The content of essential amino acids (EAAs), sweet amino acids, and bitter amino acids did not differ among the three groups in the LT.

### 3.5. DAMs Analysis

**Figure 1:**
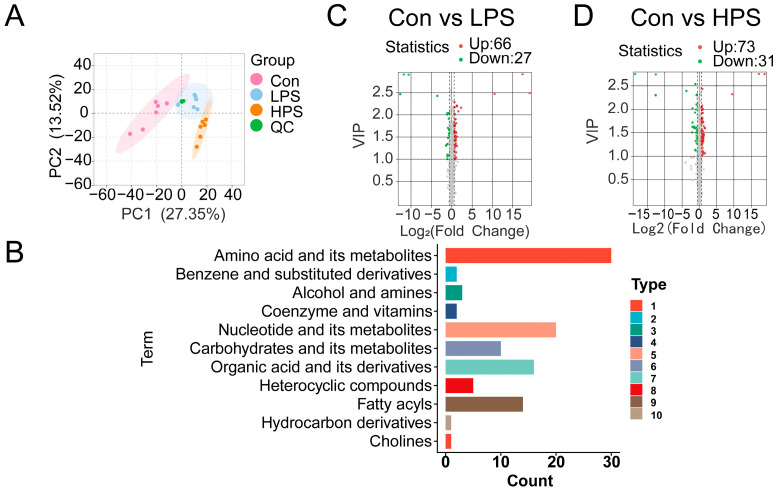
Metabolomic characteristics of *longissimus thoracis* in different treatment groups. (**A**) PCA plot of metabolome data; (**B**) classification of the DAMs; (**C**,**D**) volcano plot of DAMs.

To explore the effects of dietary supplementation with PASs on LT metabolites, their metabolic profile was analyzed using a UPLC-MS platform. Here, 897 metabolites were identified. The details of all the metabolites are shown in Supplementary Table S3. There was a clear separation among the three groups based on the PCA results (Figure 1A). Furthermore, OPLS-DA scores showed that there was significant segregation among the LPS, HPS, and CON groups, indicating that the metabolic profile of sheep muscle changed after PASs were added to the diet (Supplementary Figure S2). In this study, all DAMs were classified into 11 categories; the top 3 categories mainly including amino acids and their metabolites, nucleotides and their metabolites, and organic acids and their derivatives (Figure 1B). A total of 93 DAMs were identified, with 66 upregulated and 27 downregulated in the LPS group compared with the CON group (Figure 1C). Additionally, a total of 104 DAMs were identified in the HPS group, with 73 metabolites upregulated and 31 downregulated compared to the CON group (Figure 1D). Detailed information regarding the identified metabolites is provided in Supplementary Table S4.

**Figure 2:**
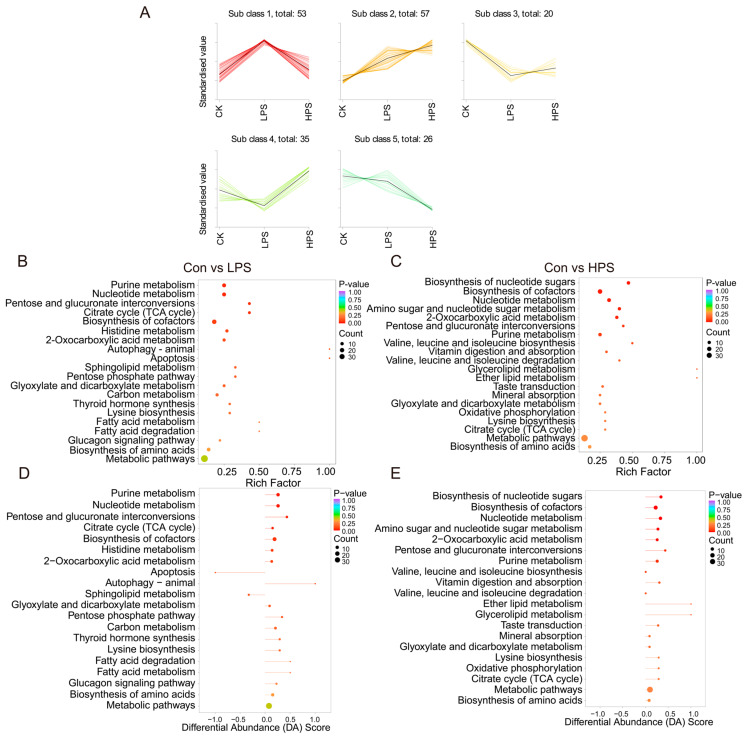
K-means classification results of DAMs and KEGG pathway enrichment analysis. (**A**) K-means analysis of DAMs; (**B**,**C**) KEGG pathway enrichment analysis of DAMs; (**D**,**E**) DAMs abundance analysis of KEGG pathways.

**Figure 3:**
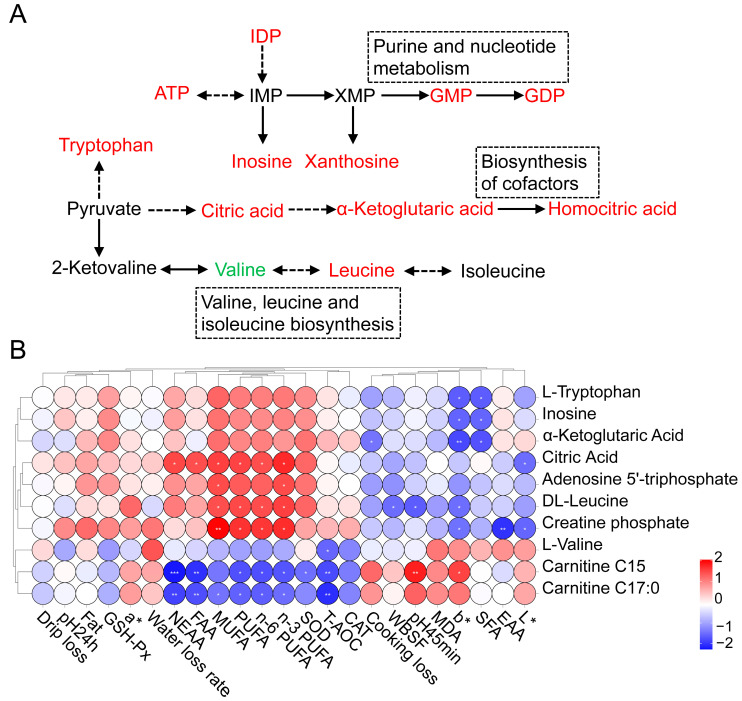
Pathway map of important metabolites and correlation analysis between DAMs and meat quality indicators. (**A**) Schematic diagram of the signal pathways. Metabolites identified are colored red, and metabolites not identified are colored black in this study. IDP: inosine 5′-diphosphate; ATP: adenosine 5′-triphosphate; IMP: inosine 5′-monophosphate; XMP: xanthosine 5′-phosphate; GMP: guanosine 5′-monophosphate; GDP: guanosine 5′-diphosphate. (**B**) DAMs and meat quality indicators heat maps. The correlations are indicated by * (0.01 < *p* < 0.05), ** (0.001 < *p* < 0.01), *** (*p ≤* 0.001), where the red squares indicate positive correlations and blue squares indicate negative correlations.

The clustering results based on different additive dosages showed five trends, among which cluster 2 showed an upward trend with an increasing dose and cluster 5 showed a downward trend with an increasing dose (Figure 2A). The metabolites enriched in clusters 2 and 5 are listed in Supplementary Table S5. The top five DAMs with the largest fold changes included creatine phosphate, 5-methoxytryptophan, guanosine-5′-diphosphate, 2-hydroxyglutaric acid, and citramalic acid in cluster 2. The top five DAMs with the largest fold changes included 7-methylguanine, L-allothreonine, and three carnitine compounds in cluster 5. Subsequently, a KEGG enrichment analysis of the DAMs was performed. Purine metabolism, nucleotide metabolism, and pentose and glucuronate interconversions were the most significant pathways in the LPS vs. CON groups. Nucleotide metabolism, cofactor biosynthesis, and nucleotide sugar biosynthesis were the most significant pathways in the HPS vs. CON groups (Figure 2B,C). Detailed information on KEGG pathway enrichment analysis is shown in Supplementary Table S6. Simultaneously, we analyzed the differential abundance scores of these enriched pathways and found that most of the metabolites enriched in these pathways were upregulated (Figure 2D,E). The important metabolites that affect muscle quality in these pathways are L-tryptophan, L-valine, citric acid, α-ketoglutaric acid, inosine, adenosine 5′-triphosphate, DL-leucine, creatine phosphate, carnitine C15, and carnitine C17:0. Based on the above enriched pathways and metabolites, we conducted a visual analysis of the muscle metabolic pathways affected by PASs (Figure 3A).

### 3.6. Analysis of the Relationship Between Major DAMs and Indicators of Meat Quality

To further explore the relationship between metabolites and meat quality, we conducted a Pearson correlation analysis between the main differential metabolites and meat quality phenotypes (Figure 3B). It can be seen from the figure that the content of various types of unsaturated fatty acids was significantly positively correlated with citric acid, DL-leucine, and creatine phosphate (r > 0.50, *p* < 0.05), but significantly negatively correlated with carnitine (r < −0.50, *p* < 0.05). Some meat quality parameters were significantly negatively correlated with L-tryptophan, inosine, α-ketoglutaric acid, and DL-leucine (r < −0.50, *p* < 0.05), but positively correlated with carnitine (r > 0.50, *p* < 0.05). NEAA and FAA were negatively correlated with carnitine but positively correlated with citric acid (r < −0.50, *p* < 0.05). T-AOC was negatively correlated with L-valine and carnitine (r < −0.50, *p* < 0.05).

## 4. Discussion

The nutritional composition of meat is affected by such factors as animal varieties, feeding methods, and nutritional composition, and within this field nutritional regulation is a vital way to improve the nutritional characteristics of meat. Intramuscular fat is closely related to the sensory quality of lamb meat, and largely determines the tenderness, juiciness, and flavor of the meat. In the present study, the protein, collagen, ash, and moisture in lamb meat did not change after adding PASs to the diet. At present, studies have shown that adding flavonoids or flavonoid-rich sea buckthorn pomace to the diet of sheep increases the fat content in the muscle [18,19]. Interestingly, we observed a significant fat content increase in the LPS group. Given the results above, we hypothesize that flavonoids in PASs may improve muscle juiciness and tenderness by increasing the crude fat content in the muscle.

It is known that cooking loss is related to the water-holding capacity (WHC) of meat. Recent studies have shown that adding *Allium mongolicum* Regel flavonoids to the sheep diet and adding rosemary extract to the broiler diet increased and decreased the cooking loss of muscle, respectively [18,20]. In this study, the cooking loss of the HPS group was reduced after adding PASs to the diet, which was inconsistent with the above results. Studies have shown that the supplementation of natural antioxidant vitamin E can improve the WHC of muscle, which may affect the length of the sarcomere and the integrity of the membrane [21,22]. The difference in our results may be due to the different antioxidant properties of flavonoids and phenolic compounds in PASs. Meat color can be used as a reference standard to judge the freshness of meat, which is one of the key factors affecting consumer acceptance. Meat color is affected by myoglobin, which is easily oxidized by O_2_ to form bright red oxymyoglobin, which can be further oxidized to form brown metmyoglobin. The supplementation of dietary oils with antioxidants can significantly increase the concentration of oxymyoglobin and decrease the concentration of metmyoglobin in meat, thereby improving the meat color [23]. In this study, the lamb meat in the LPS and HPS groups showed lower L* and b* pH 45 minvalues. We speculate that the flavonoids in PASs may increase the antioxidant performance of muscle and prevent the conversion of oxymyoglobin to metmyoglobin, thereby improving the meat color. The above results show that the addition of PASs to the diet had a positive effect on meat quality.

Oxidative stress arises from excessive reactive oxygen species (ROS) production, which leads to an imbalance in the body’s endogenous protection mechanism [24]. Therefore, the body’s antioxidant defense system will defend cells and tissues from oxidative injury through a series of antioxidant substances and enzymes such as SOD, GSH-Px, CAT, and T-AOC, which are closely related to animal health [4]. MDA is a lipid peroxidation product induced by oxygen free radicals, and its content reflects oxidative damage in cells. Plant active ingredients such as flavonoids, saponins, and polysaccharides can scavenge free radicals and play an antioxidant role [25,26]. The addition of flavonoids to the diet can reduce the MDA content in the plasma of lamb meat and enhance the body’s antioxidant capacity [27]. In the present study, the SOD, CAT, GSH-Px, and T-AOC in serum increased to varying degrees after adding PASs to the diet, but the content of MDA was decreased. We speculate that PASs may enhance the body’s antioxidant capacity. Meat quality is largely related to the level of muscle oxidation, which depends on the antioxidant content or activity. Flavonoids and phenolic compounds are bioactive components isolated from plants, and they not only have a healthcare effect but also inhibit the oxidation and deterioration of oil [26]. Interestingly, phenolic compounds in dried pomace can regulate the oxidation levels of muscle [28]. The addition of flavonoids or phenolic compounds to the diet has been found to regulate the oxidation level of muscles and reduce MDA content in the plasma of lamb meat [27]. In the present study, the T-AOC, CAT, and GSH-Px in muscle were significantly increased, but the content of MDA was significantly decreased in the HPS group. One explanation for the enhancement of muscle antioxidant capacity by PASs may be that flavonoids and phenolic compounds in PASs protect cells from oxidative damage by scavenging free radicals or inhibiting metal-ion-catalyzed oxidation reactions [29,30]. Another possibility is that the supplementation of PASs in the diet enhances the expression of endogenous antioxidant systems by activating the Nrf2-Keap1 pathway [31,32], which reduces lipid peroxidation. Overall, our results showed that dietary PAS supplementation enhanced the antioxidant capacity of lamb meat, with the HPS group having the greatest effect.

The odor of lamb meat is closely related to some volatile FAs, especially branched chains and unsaturated FAs with 8–10 carbon atoms. These FAs are critical flavor precursors in lamb meat, significantly affecting flavor characteristics of lamb through chemical reactions to produce volatile compounds (e.g., aldehydes, ketones, and alcohols). In this study, the contents of C8:0 and C10:0 were significantly reduced after the addition of high doses of PASs, indicating that the adding of PASs has an active effect on improving the smell of lamb meat. Reducing SFA content and increasing PUFA content in lamb meat can improve its nutritional value. Replacing SFA and trans-FAs with MUFA can reduce coronary heart disease risk [33]. The trend of SFA and PUFA in meat with the addition of PASs is in line with the demand for healthy food. PASs contain active ingredients that have strong antioxidant capacity and inhibit polyunsaturated FA biohydrogenation by regulating changes in rumen microbial flora, resulting in excess polyunsaturated FAs being absorbed in the rumen and deposited in the muscle [34], which is a possible reason for the increase in PUFA in the PAS addition group. N-3 PUFA inhibits inflammation occurrence through the NOD-like receptor signaling pathway; therefore, increasing n-3 PUFA intake reduces certain chronic disease risks [35]. EPA and DHA are essential components of n-3 PUFA and FAs in the human body. They cannot be synthesized in the body and must be ingested through the diet. C18:3n3 is essential for EPA and DHA synthesis and has critical biological functions in the human body (e.g., antioxidant, inflammatory regulation, immune regulation, and cardiovascular health activities) [36]. In our study, the increase in C18:3n3 content may promote the synthesis of EPA and DHA, increasing the nutritional value of lamb meat. The addition of polyphenol-rich grape pomace to fattening cattle diets increased the n-3 PUFA content in the LT [31]. This finding is consistent with those of this study; thus, phenolic substances in PASs and grape pomace may play a similar functional role in n-3 PUFA production. The composition of beneficial FAs has a vital effect on lamb meat quality, and the PUFA/SFA ratio is closely related to the nutritional value of lamb meat. Dietary supplementation with flavonoid-rich compounds, such as hesperidin and naringin, increased PUFA content and the PUFA/SFA ratio in chickens [37]. Zhao et al. [38] observed that dietary *Allium mongolicum* Regel ethanol extract supplementation increased n-3 PUFAs and SFA content, but had no significant effect on the PUFA/SFA ratio. Interestingly, in the present study, the PUFA content and PUFA/SFA ratio were increased after adding PASs to the diet. The reason for the inconsistency of the above results may be related to the different species or the different content of phenols and flavonoid compounds. Therefore, the addition of PASs to the diet of sheep can effectively improve the composition of FAs in the muscle and regulate lipid metabolism, which may be related to the phenols and flavonoid compounds contained in PASs.

The type and content of amino acids in muscle and the composition ratio of various EAAs affect meat quality. Flavor amino acids are chemical substances with an umami taste, which are mainly composed of aspartate, glycine, arginine, and glutamic acid [39]. Glycine and glutamic acid can react with reducing sugars through the Meladic reaction to produce aldehydes, ketones, esters, and alcohols, which provide a unique flavor in lamb meat. Glycine and glutamic acid are taste stimulators and neuromodulators [40], and their interaction with taste buds is the main factor affecting the perception of umami [41,42]. In addition, amino acids are involved in muscle protein synthesis and breakdown. Amino acids are divided into EAA and NEAA based on whether the human body can synthesize them by itself. In the current study, the results showed that the contents of flavor amino acids (aspartate, glycine, arginine, and glutamic acid) and EAA (tryptophan and threonine) in muscle were significantly increased after the addition of PASs rich in flavonoids and phenols to the diet. This result is similar to those of the dietary supplementation of *Allium mongolicum* Regel extracts rich in flavonoids and phenols, which improved the amino acid structure and flavor of the lamb meat [43].

As important metabolic molecules in muscle, amino acids and their derivatives have an important influence on the quality of muscle. The increase in tryptophan levels may lead to an increase in its metabolite, indole-3-propionic acid (IPA), which is known for its antioxidant, anti-inflammatory, and antibacterial activities [44]. From this, we may speculate that the increase in tryptophan content may indirectly increase the antioxidant properties of muscle. Leucine regulates protein synthesis in the dorsal muscle in an mTOR-dependent manner, thereby affecting meat quality [45]. In this study, the observed increase in muscle DL-leucine content following PAS supplementation suggests its potential role in regulating muscle protein synthesis and subsequently its influence on meat quality. Metabolites in the nucleotide metabolic pathway, such as inosine monophosphate (IMP), guanosine monophosphate (GMP), and inosine, are involved in the energy metabolism of muscles and contribute to the formation of muscle umami. The ATP in muscle tissues can directly affect the force and duration of muscle contraction, which can affect meat tenderness and texture [46]. ATP can be catalyzed by adenylate cyclase to 3′,5′-cyclic adenosine monophosphate, which in turn produces AMP under the action of high affinity cAMP-specific 3′, 5′-cyclic phosphodiesterase 7. AMP can be further degraded to produce inosine monophosphate and inosine, which affect meat flavor. AMP can not only regulate lipid metabolism in muscle but also be used as a flavoring regulator to improve meat quality [47]. In the present study, after the addition of PASs, the degradation products of ATP were continuously generated in lamb meat, which ultimately had a positive effect on meat flavor. In addition, the citric acid content in the biosynthesis of cofactors was also significantly increased, and this is not only one of the key intermediates in the regulation of the glycolytic pathway and TCA cycle but also a component of the energy metabolism pathway. Lactic acid, ATP, and other metabolites produced by the glycolysis pathway can affect the taste and flavor characteristics of muscle. Given the above results, we speculate that the addition of PASs may improve muscle quality by regulating the glycolysis pathway and TCA cycle. Creatine phosphate, as a high-energy compound, is a rapid regeneration source of ATP in muscle. When the levels of ATP and creatine phosphatein are high in the muscle, the muscle can undergo glycolysis faster during post-slaughter maturation, which contributes to the softening of the muscle tissue and may improve the tenderness of the meat [48,49]. Carnitine and acylcarnitine derivatives play an essential role in fatty acid oxidation, thus indirectly affecting the flavor of meat. In this study, the content of carnitine compounds in the muscle decreased after adding PASs to the diet, and this may have led to an increase in fatty acid oxidation and may ultimately affect meat quality. An intermediate product of the TCA cycle, α-ketoglutaric acid, can be converted into L-glutamic acid. In meat products, glutamic acid exists in two forms, free and bound, which critically influence meat flavor and quality. However, the direct effect of α-ketoglutaric acid on meat quality is unknown but might affect meat quality by mutual transformation with glutamic acid.

The content and proportion of metabolites in muscle will directly or indirectly affect the texture, taste, color, and nutritional value of muscle. The integrated analysis of muscle metabolites and meat quality parameters may help to determine the potential effects of specific metabolites on meat quality. In this study, we focused on the differential metabolites in the main metabolic pathways to explore their relationship with meat quality parameters. Citric acid, as an important intermediate product in the TCA cycle, can be decomposed into oxaloacetate and acetyl-CoA, which can be used for the synthesis of fatty acids. Acetyl-CoA-carboxylase is the rate-limiting enzyme in the fatty acid synthesis pathway, and is responsible for the conversion of acetyl-CoA to malonyl-CoA. Citric acid is an allosteric activator of acetyl-CoA-carboxylase, and indicates that the presence of citric acid can promote the activity of acetyl-CoA-carboxylase and accelerate the first step of fatty acid synthesis. In this study, the contents of citric acid and unsaturated fatty acids increased after the addition of PASs to the diet, which may explain the significant positive correlation between the two in lamb meat. Leucine was negatively correlated with shear force in pork and pH 24 h after slaughter but showed no significant correlation with meat color [50]. Our results showed a significant negative correlation between leucine and shear force, pH 45 min, and b* in lamb meat, which was partially consistent with the above results. This may be attributed to different feeding methods for lamb meat and different determination times for meat color and pH. Peukert et al. found that inosine was negatively correlated with meat color in pork [51], and we obtained similar results in lamb meat. There is no direct evidence of a correlation between other key metabolites and meat quality parameters. These results may be related to energy metabolism and fatty acid oxidation in muscles after slaughter, and may warrant further experimental verification.

## 5. Conclusions

In this study, the effects of PAS addition to the diet on the meat quality and metabolites of lamb meat were analyzed. Meat quality analysis revealed that the addition of PASs could increase the intramuscular fat content of the LT muscle and reduce muscle lightness, yellowness, and cooking loss. Additionally, PAS supplementation could improve the composition of flavor amino acids, reduce the deposition of SFA, promote the deposition of MUFA and PUFA, and enhance the muscle’s antioxidant capacity. The main pathways of LT metabolism affected by diet were purine metabolism, nucleotide metabolism, and biosynthesis of cofactors. Among them, tryptophan, leucine, citric acid, adenosine 5′-triphosphate (ATP), creatine phosphate, inosine, and α-ketoglutaric acid were the key metabolites affecting meat quality. Notably, supplementation with 6% of PASs had a greater impact on lamb meat quality in this study, although further research is needed to confirm the effects of PAS levels on lamb meat quality. Given the biological function of PASs and the results obtained in this study, we believe that PASs can be used as a feed component in lamb production to improve meat quality and muscle antioxidant properties.

## Figures and Tables

**Table 1:** Effects of dietary PAS supplementation on meat physicochemical characteristics of Hu lambs.

Parameters	Group ^1^				*p*-Value
	CON	LPS	HPS	SEM	
Moisture (%)	73.03	72.91	71.89	0.234	0.087
Ashes (%)	0.99	0.92	0.95	0.022	0.471
Collagen (%)	0.91	0.82	0.76	0.039	0.306
Fat (%)	2.02 ^b^	2.08 ^b^	2.59 ^a^	0.103	0.031
Protein (%)	21.09	21.63	22.24	0.241	0.148
Drip loss, %	5.36	4.74	4.07	0.502	0.605
Water loss rate, %	7.23	7.54	7.03	0.454	0.909
Cooking loss, %	26.58 ^a^	22.41 ^ab^	19.19 ^b^	1.278	0.049
Shear force (N) ^2^	48.89	46.25	44.46	1.766	0.616
Lightness (L*) 45 min	31.3 ^a^	28.2 ^b^	28.7 ^b^	0.458	0.004
Redness (a*) 45 min	18.7	19.7	19.1	0.245	0.291
Yellowness (b*) 45 min	9.3 ^a^	7.5 ^b^	7.0 ^b^	0.285	<0.01
pH _45min_	6.34	6.38	6.25	0.040	0.434
pH _24h_	5.57	5.47	5.65	0.043	0.268

^a,b^ means within a row with different superscript letters are significantly different (*p* < 0.05). ^1^ CON, basal diet; LPS, CON with 3% PAS, low-dose PAS; HPS, CON with 6% PAS, high-dose PAS; SEM, standard error of mean. ^2^ WBSF, Warner–Bratzler shear force.

**Table 2:** Effects of dietary PAS supplementation on the antioxidation capacity and MDA content of Hu lambs’ serum and longissimus thoracis.

Items ^2^	Group ^1^				*p*-Value
	CON	LPS	HPS	SEM	
Serum					
T-AOC, U/mL	9.07 ^c^	10.15 ^b^	11.93 ^a^	0.313	<0.01
SOD, U/mL	37.56 ^b^	41.00 ^b^	47.49 ^a^	1.267	<0.01
GSH-Px, U/mL	209.08 ^b^	224.57 ^ab^	241.96 ^a^	5.831	0.046
CAT, U/mL	35.87 ^c^	42.42 ^b^	46.62 ^a^	1.649	<0.01
MDA, U/mg. protein	0.58 ^a^	0.52 ^ab^	0.43 ^b^	0.025	0.040
*Longissimus thoracis*					
T-AOC, U/mg. protein	1.74 ^b^	2.11 ^ab^	2.60 ^a^	0.141	0.033
SOD, U/mg. protein	14.38	15.89	19.08	0.866	0.068
GSH-Px, U/mg. protein	41.44 ^b^	44.64 ^b^	52.02 ^a^	1.361	<0.01
CAT, U/mg. protein	4.37 ^b^	4.96 ^b^	6.03 ^a^	0.246	0.010
MDA, U/mg. protein	0.58 ^a^	0.52 ^ab^	0.43 ^b^	0.025	0.040

^a,b,c^ means within a row with different superscript letters are significantly different (*p* < 0.05). ^1^ CON, basal diet; LPS, CON with 3% PAS, low-dose PAS; HPS, CON with 6% PAS, high-dose PAS; SEM, standard error of mean. ^2^ T-AOC, total antioxidant capacity; SOD, superoxide dismutase; GSH-Px, glutathione peroxidase; CAT, catalase; MDA, malondialdehyde.

**Table 3:** Effects of dietary PAS supplementation on the content of fatty acids of Hu lambs (mg/100 g meat).

Items ^2^	Group ^1^				*p*-Value
	CON	LPS	HPS	SEM	
C8:0	1.68 ^a^	1.51 ^ab^	1.37 ^b^	0.046	0.009
C9:0	0.57	0.53	0.58	0.021	0.601
C10:0	8.06 ^a^	6.84 ^ab^	6.19 ^b^	0.298	0.022
C11:0	0.26 ^a^	0.21 ^b^	0.20 ^b^	0.011	0.047
C12:0	5.04	5.41	6.61	0.392	0.245
C13:0	13.61	12.68	13.83	0.485	0.619
C14:0	70.42	70.51	69.64	1.654	0.975
C15:0	8.47	7.76	7.27	0.270	0.197
C16:0	554.94	523.72	526.32	6.022	0.053
C17:0	23.97 ^a^	20.23 ^b^	19.51 ^b^	0.762	0.025
C18:0	534.39	507.40	514.66	5.205	0.083
C19:0	3.36	3.43	3.68	0.174	0.748
C20:0	2.55	2.51	2.61	0.068	0.860
C21:0	0.26	0.24	0.24	0.004	0.440
C22:0	0.59	0.60	0.60	0.018	0.960
C24:0	0.38	0.38	0.39	0.009	0.750
C14:1	3.83 ^a^	3.02 ^b^	2.94 ^b^	0.154	0.020
C15:1	3.20 ^a^	2.28 ^b^	2.31 ^b^	0.180	0.049
C16:1	50.41	54.24	56.42	1.122	0.078
C18:1n9c	562.46 ^c^	616.07 ^b^	651.18 ^a^	9.893	<0.01
C19:1(cis:10)	3.89	3.51	3.50	0.087	0.118
C20:1(cis:11)	3.73	3.62	3.72	0.107	0.911
C22:1n9	2.26 ^a^	1.84 ^b^	1.84 ^b^	0.080	0.030
C22:1T	0.37 ^a^	0.26 ^b^	0.32 ^ab^	0.019	0.038
C18:2n6c	56.38 ^c^	71.44 ^b^	88.94 ^a^	3.252	<0.01
C18:2n6t	8.39 ^c^	10.43 ^b^	14.82 ^a^	0.673	<0.01
C18:3n3	16.87 ^c^	19.62 ^b^	23.32 ^a^	0.770	<0.01
C18:3n6	2.09	2.41	2.74	0.169	0.310
C20:3n3	10.76 ^c^	12.79 ^b^	14.25 ^a^	0.425	<0.01
C20:3n6	10.61	10.89	10.49	0.465	0.943
C20:4n6	17.40	18.42	18.82	0.348	0.236
C20:5n3	1.25	1.30	1.56	0.101	0.441
C22:6n3	7.89 ^b^	8.70 ^ab^	10.26 ^a^	0.368	0.017
SFA	1228.58 ^a^	1163.98 ^b^	1173.715 ^b^	11.811	0.044
MUFA	630.15 ^c^	684.83 ^b^	722.23 ^a^	10.518	<0.01
PUFA	131.64 ^c^	156.00 ^b^	185.20 ^a^	5.528	<0.01
n-3 PUFA	36.78 ^c^	42.41 ^b^	49.37 ^a^	1.369	<0.01
n-6 PUFA	94.87 ^b^	113.58 ^b^	135.82 ^a^	4.191	<0.01
n-6/n-3	2.58 ^b^	2.68 ^ab^	2.75 ^a^	0.026	0.018
PUFA/SFA	0.11 ^c^	0.13 ^b^	0.16 ^a^	0.005	<0.01
TFA	1990.37 ^b^	2004.81 ^b^	2081.15 ^a^	16.65	0.046

^a,b,c^ mean values with different superscript letters were significantly different (*p* < 0.05). ^1^ CON, basal diet; LPS, CON with 3% PAS, low-dose PAS; HPS, CON with 6% PAS, high-dose PAS; SEM, standard error of mean. ^2^ SFA = sum of C8:0, C9:0, C10:0, C11:0, C12:0, C13:0, C14:0, C15:0, C16:0, C17:0, C18:0, C19:0, C20:0, C21:0, C22:0, and C24:0; MUFA = sum of C14:1, C15:1, C16:1, C18:1n9c, C19:1(cis:10), C20:1(cis:11), C22:1n9, and C22:1T; PUFA = sum of C18:2n6c, C18:2n6t, C18:3n3, C18:3n6, C20:3n3, C20:3n6, C20:4n6, C20:5n3, and C22:6n3; n-3 PUFA = sum of C18:3n3, C20:3n3, C20:5n3, and C22:6n3; n-6 PUFA = sum of C18:2n6c, C18:2n6t, C18:3n6, C20:3n6, and C20:4n6; TFA = total fatty acids.

**Table 4:** Effects of dietary PAS supplementation on the content of amino acids in Hu lambs.

Amino Acids (g/100g) ^2^	Group ^1^				*p*-Value
	CON	LPS	HPS	SEM	
Valine	0.72	0.69	0.73	0.037	0.941
Isoleucine	0.57	0.63	0.53	0.022	0.224
Leucine	1.27	1.19	1.27	0.035	0.643
Phenylalanine	0.47	0.47	0.44	0.011	0.542
Methionine	0.23	0.23	0.23	0.007	1.000
Tryptophan	1.73 ^b^	1.75 ^b^	1.96 ^a^	0.040	0.022
Threonine	0.79 ^b^	0.86 ^a^	0.86 ^a^	0.008	<0.01
Lysine	0.74	0.74	0.61	0.040	0.352
Tyrosine	0.82	0.78	0.81	0.017	0.697
Aspartate	1.74 ^b^	1.71 ^b^	2.16 ^a^	0.076	0.018
Glycine	0.91 ^b^	0.96 ^b^	1.05 ^a^	0.018	<0.01
Arginine	0.92 ^b^	0.95 ^b^	1.31 ^a^	0.073	0.040
Histidine	0.48 ^a^	0.35 ^b^	0.42 ^ab^	0.017	0.005
Alanine	1.62	1.49	1.52	0.062	0.687
Serine	0.36	0.31	0.33	0.010	0.119
Asparagine Anhydrous	1.12	0.97	1.00	0.062	0.624
Proline	0.31 ^b^	0.41 ^a^	0.42 ^a^	0.075	0.018
Glutamic acid	1.70 ^b^	1.72 ^b^	2.19 ^a^	0.077	0.007
Glutamine	2.97	3.00	2.97	0.097	0.990
EAA	6.54	6.76	6.92	0.148	0.606
NEAA	13.21 ^c^	14.26 ^b^	15.61 ^a^	0.297	0.001
FAA	5.92 ^c^	6.71 ^b^	7.43 ^a^	0.188	<0.01
SAA	4.72	4.84	5.11	0.099	0.314
BAA	7.04	6.96	7.20	0.159	0.840
TAA	19.48	19.24	20.80	0.310	0.080

^a,b,c^ mean values with different superscript letters were significantly different (*p* < 0.05). ^1^ CON, basal diet; LPS, CON with 3% PAS, low-dose PAS; HPS, CON with 6% PAS, high-dose PAS; SEM, standard error of mean. ^2^ EAA = sum of valine, isoleucine, leucine, phenylalanine, methionine, tryptophan, threonine, and lysine; NEAA = sum of tyrosine, aspartate, glycine, arginine, histidine, alanine, serine, asparagine anhydrous, proline, glutamic acid, and glutamine; FAA = sum of aspartate, glycine, alanine, phenylalanine, and glutamic acid; SAA = sum of threonine, lysine, glycine, serine, alanine, and proline; BAA = sum of histidine, valine, methionine, isoleucine, leucine, phenylalanine, tyrosine, tryptophan, and arginine; TAA = total amino acids.

## Data Availability

The original contributions presented in the study are included in the article/Supplementary Materials, further inquiries can be directed to the corresponding author.

## Supplementary Materials

The following supporting information can be downloaded at: https://www.mdpi.com/article/10.3390/foods13213415/s1, Figure S1: Classification of bioactive substances in prickly ash seeds; Figure S2: OPLS-DA score plots and OPLS-DA permutation test of *longissimus thoracis* metabolic profiling; Table S1: Content of conventional nutrients in prickly ash seeds; Table S2: Composition and nutrient levels of basal diets (DM Basis); Table S3: Details of all the metabolites in each group; Table S4: Details for DAMs in Con vs. LPS and Con vs. HPS groups; Table S5: Details for DAMs in cluster 2 and cluster 5; Table S6: Detailed information about the KEGG enrichment analysis of the DAMs in Con vs. LPS and Con vs. HPS.
