# Supplementary material for: Effects of Prickly Ash Seed Dietary Supplementation on Meat Quality, Antioxidative Capability, and Metabolite Characteristics of Hu Lambs
Source: Foods. 2024 Oct 26;13(21):3415. doi: 10.3390/foods13213415 (PMC11545103; doi:10.3390/foods13213415)
Supplement: Supplementary file 1 [file foods-13-03415-s001.zip › Supplementary Figure.pdf]

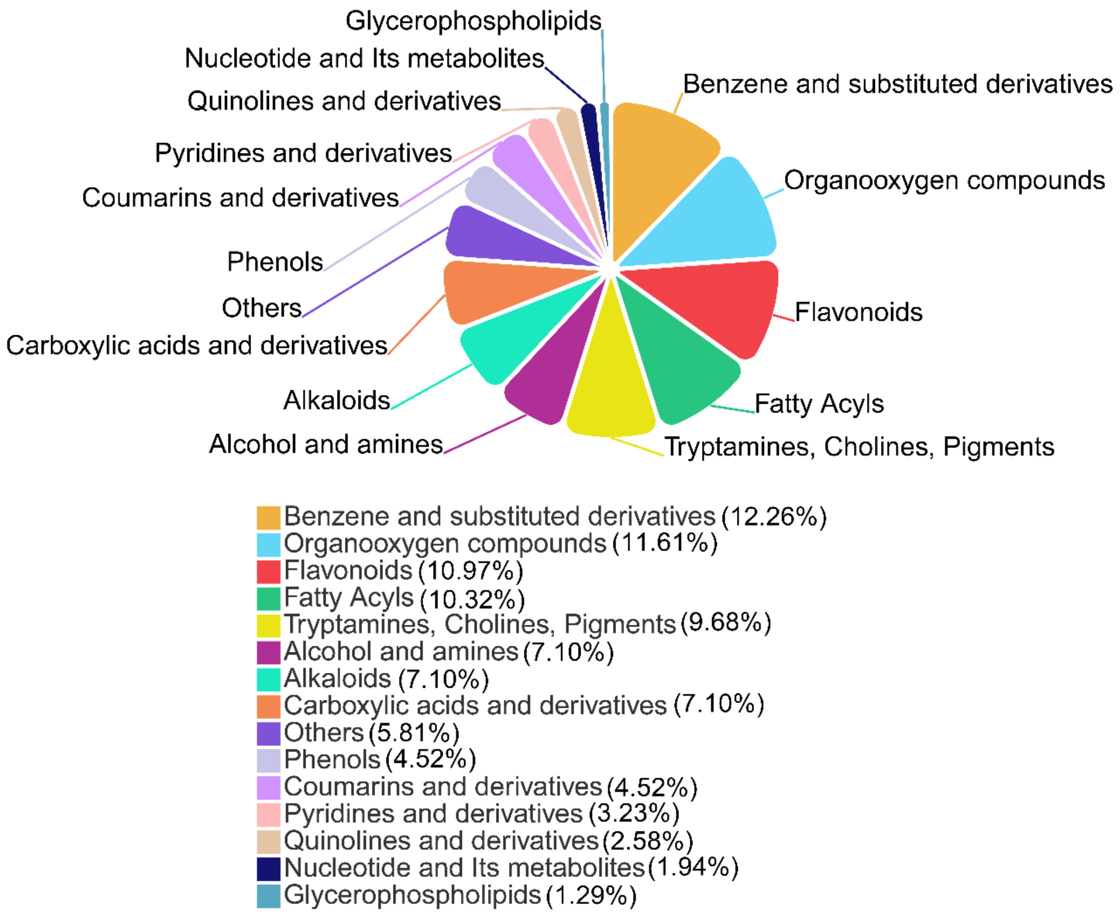

Figure S1. Classification of bioactive substances in prickly ash seeds

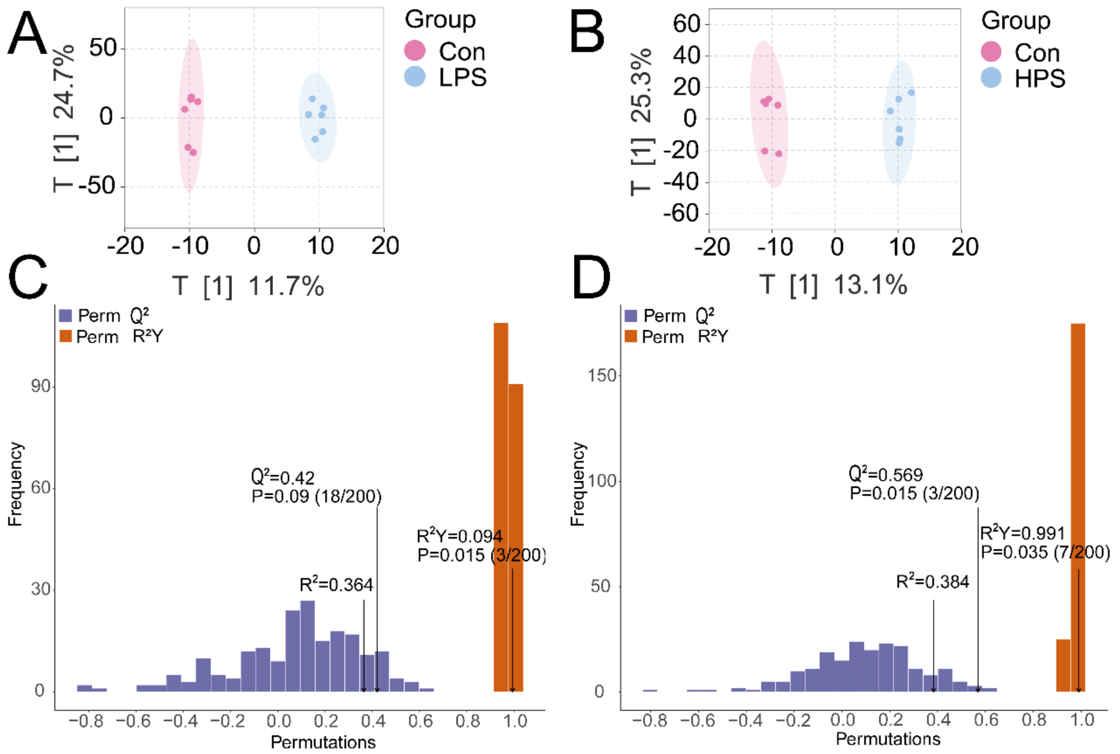

Figure S2. OPLS-DA score plots and OPLS-DA permutation test of longissimus thoracis metabolic profiling. (a, b) OPLS-DA score plots; (c, d) Permutation tests of OPLS-DA
